# Cardiomiopatia Dilatada: Nova Variante no Gene da Filamina-C

**DOI:** 10.36660/abc.20200199

**Published:** 2021-07-14

**Authors:** Murilo Zomer Frasson, Cristiano Pederneiras Jaeger

**Affiliations:** 1 Hospital Mãe de Deus Porto AlegreRS Brasil Hospital Mãe de Deus - Residência Médica de Cardiologia, Porto Alegre, RS - Brasil.

**Keywords:** Doenças Cardiovasculares/fisiopatologia, Cardiomiopatia Dilatada/genética, Insuficiência Cardíaca, Filaminas

## Relato de Caso

Apresentamos o relato de caso de um paciente masculino de 50 anos de idade que procurou o departamento de pronto-socorro de nossa instituição com queixa de dispneia progressiva há 1 semana. O paciente relatava história prévia de fibromialgia e negava demais comorbidades, como infarto miocárdico ou acidente vascular cerebral. Negava tabagismo atual e fazia uso eventual de álcool. Como medicamentos de uso contínuo, utilizava duloxetina, pregabalina e zolpidem. Mãe e primos com história de insuficiência cardíaca por miocardiopatia dilatada idiopática, sem demais comorbidades cardiovasculares em seu histórico familiar.

**Figura 1:**
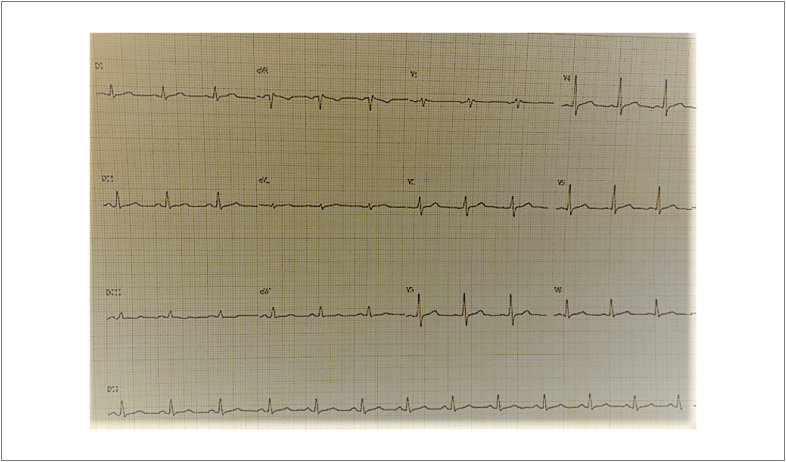
Eletrocardiograma.

Ao exame físico, apresentava sinais vitais estáveis, crepitações em bases pulmonares e edema de membros inferiores. Ausculta cardíaca sem comemorativos. Eletrocardiograma realizado mostrava ritmo sinusal, sem sinais de sobrecarga atrial ou ventricular (Figura 1). Radiografia de tórax revelou cardiomegalia. Nos exames laboratoriais, chamava a atenção um NT-pró-BNP de 2.335 pg/mL, e a série de troponinas T ultrassensíveis revelou valores consecutivos de 0,074 ng/mL e 0,072 ng/mL (valor de referência <0,014 ng/mL).

O paciente foi internado para investigação. Ecocardiograma revelou uma cardiopatia dilatada com fração de ejeção de 37,1%, com uma hipocinesia difusa das paredes do ventrículo esquerdo. Um cateterismo cardíaco revelou coronárias sem estenoses significativas. Foi realizada ressonância magnética cardíaca, que demonstrou fração de ejeção de 27% com disfunção sistólica global e ausência de fibrose, sugerindo cardiomiopatia dilatada idiopática. Também foi realizado *holter* de 24 horas, que não evidenciou arritmias ventriculares durante o período de monitoramento.

Após compensação com medidas para insuficiência cardíaca na instituição, o paciente realizou teste genético para cardiomiopatias hereditárias, tendo sido analisados os genes listados na Tabela 1. A análise foi feita com extração e fragmentação do DNA genômico, seguida de identificação, captura e enriquecimento das regiões de interesse. O resultado de tal exame revelou uma variante provavelmente patogênica c.1595delIT em heterozigose no gene da filamina-C (*FLNC*). Este gene tem sido associado a uma série de cardiopatias, tais como: (1) cardiomiopatia hipertrófica familiar, de herança não determinada; (2) cardiomiopatia restritiva familiar,^1^ de herança autossômica dominante [OMIM: 617047]; (3) miopatia distal de herança autossômica dominante [OMIM: 614065]; e (4) miopatia miofibrilar, de herança autossômica dominante [OMIM: 609524].^2^ A variante identificada é caracterizada pela deleção de um nucleotídio que, preditamente, leva à alteração da matriz de leitura (*frameshift*) ao promover a substituição do aminoácido valina no códon 532 por uma glicina, com consequente parada precoce da tradução proteica 16 posições à frente (p.(Val532Glyfs*16)) resultando em uma proteína truncada. A variante está ausente nos bancos de frequência populacionais (Exome Aggregation Consortium [ExAC] e The Genome Aggregation Database [GnomAD]),^3^ não foi descrita na literatura médica em nenhum momento e nunca foi observada no banco de dados ClinVar.^4^ A filamina-C é uma proteína expressada principalmente no músculo cardíaco e músculo esquelético, sendo codificada pelo gene *FLNC*. A proteína é responsável pelo *crosslink* dos filamentos de actina em redes ortogonais no citoplasma cortical das células e participa da ancoragem de proteínas de membrana para o citoesqueleto da actina.^5^ Devido a essas funções e sua expressão no músculo cardíaco predominante, o gene *FLNC* tem sido relacionado a cardiomiopatias dilatadas ou arritmogênicas.^6^ Segundo as métricas disponíveis no banco de dados GnomAD, o gene *FLNC* não tolera alterações *loss-of-function*. Ademais, mutações *frameshift* têm sido relacionadas às doenças de cardiomiopatia arritmogênica/dilatada.^7^ De acordo com os critérios do American College of Medical Genetics and Genomics (ACMG),^8^ esta variante encontrada é classificada como provavelmente patogênica.

**Tabela 1:** Painel genético analisado

ABCC9; ACTC1; ACTN2; ANK2; BAG3; BRAF; CALR3; CAV3; CBL; CRYAB; CSRP3; DES; DSC2; DSG2; DSP; DTNA; EMD; EYA4; FHL1; FKTN; FLNC; GAA; GLA; HRAS; JPH2; JUP; KRAS; LAMP2; LDB3; LMNA; MAP2K1; MAP2K2; MYBPC3; MYH6; MYH7; MYL2; MYL3; MYLK2; MYOT; MYOZ2; NEBL; NEXN; NRAS; PKP2; PLN; PRKAG2; PSEN1; PSEN2; PTPN11; RAF1; RBM20; RPSA; RYR2; SCN5A; SGCD; SHOC2; SLC25A4; SOS1; SPRED1; SYNE1; SYNE2; TAZ; TCAP; TGFB3; TMEM43; TMPO; TNNC1; TNNI3; TNNT2; TPM1; TRIM63; TTN; TTR; VCL.

O paciente apresentou melhora clínica com o tratamento, tendo recebido alta com furosemida, sacubitril-valsartana e carvedilol. Foi recomendado aconselhamento genético à família, posto que familiares em primeiro grau de indivíduos portadores de variantes patogênicas em heterozigose do gene *FLNC* têm 50% de probabilidade de serem portadores da mesma variante. Apesar do risco de arritmias ventriculares importantes, neste caso específico, o paciente optou pelo não implante de cardiodesfibrilador (CDI) em um primeiro momento. Quanto a esse aspecto de prevenção de morte súbita cardíaca, lembramos que o estudo DANISH^9^ não demonstrou benefício em termos de diminuição de mortalidade em pacientes com insuficiência cardíaca de fração de ejeção reduzida de etiologia não isquêmica. Contudo, é importante ressaltar que, de acordo com diretrizes da Sociedade Brasileira de Cardiologia, pacientes com insuficiência cardíaca de etiologia não isquêmica com fração de ejeção ≤35% poderão ter indicação de CDI, inclusive para profilaxia primária (Classe IIa).^10^ Além disso, as diretrizes do Heart Rhythm Journal de 2019^11^ citam que, em pacientes com cardiomiopatia arritmogênica ligada a mutação no gene *FLNC* e fração de ejeção <45%, o implante do CDI é terapia a ser considerada (Classe IIa/C). Esta seria uma recomendação mais específica, visto que leva em consideração o caráter genético da patologia do paciente. Sentimos que a decisão quanto ao implante ou não de CDI deva levar em conta as evidências literárias, mas que também seja individualizada, observando-se sempre a vontade do paciente e sua qualidade e expectativa de vida.

Diante de tal relato, consideramos a importância de se buscar ativamente a etiologia para casos de insuficiência cardíaca presumidamente idiopática, incluindo a pesquisa genética, visto que há a possibilidade de que seja encontrado um fator causal para a doença de um paciente, que muitas vezes pode estar sendo privado de um diagnóstico mais específico. Evidentemente, existem certas dificuldades para a oferta do rastreamento genético, tais como baixa disponibilidade do exame em muitos locais, preço elevado e falta de difusão do conhecimento genético entre os cardiologistas gerais. Salienta-se que a genética é um campo ainda em grande desenvolvimento, no qual certamente muitas mutações e variantes patogênicas ainda devem ser catalogadas, possibilitando orientações muito mais precisas ao paciente e seus familiares, uma vez determinada a origem da patologia em questão.
