# Supplementary material for: Cardiomiopatia Dilatada: Nova Variante no Gene da Filamina-C
Source: Arq Bras Cardiol. 2021 Jul 14;117(1 Supl 1):16–8. [Article in Portuguese] doi: 10.36660/abc.20200199 (PMC8291900; doi:10.36660/abc.20200199)
Supplement: Translation

# Translation: Dilated Cardiomyopathy: New Variant in the Filamin-C Gene

**Authors:** Murilo Zomer Frasson, Cristiano Pederneiras Jaeger

**Affiliations:** 1 Porto AlegreRS Brazil Hospital Mãe de Deus - Residência Médica de Cardiologia, Porto Alegre, RS - Brazil.

**Keywords:** Cardiovascular Diseases/physiopathology, Cardiomyopathy Dilated/genetics, Heart Failure, Filamins

## Case Report

This is a case report of a 50-year-old male patient who came to the emergency department of our institution with a complaint of progressive dyspnea for a week. The patient reported a previous history of fibromyalgia and denied other comorbidities, such as myocardial infarction or stroke. He denied current smoking, but occasionally used alcohol. His routine medications included duloxetine, pregabalin and zolpidem. His mother and cousins had a history of heart failure due to idiopathic dilated cardiomyopathy, with no other cardiovascular comorbidities in his family history.

On physical examination, he showed stable vital signs, crackles in the lung bases and edema in the lower limbs. Cardiac auscultation showed normal heart sounds. The electrocardiogram showed sinus rhythm, with no signs of atrial or ventricular overload (Figure 1). The chest radiography revealed cardiomegaly. The laboratory tests showed an NT-pro-BNP of 2335 pg/mL, and the series of ultrasensitive T-troponins revealed consecutive values of 0.074 ng/mL and 0.072 ng/mL (reference value < 0.014 ng/mL).

**Figure 1:** Electrocardiogram.

The patient was admitted for investigation. His echocardiogram revealed dilated heart disease, an ejection fraction of 37.1%, with diffuse hypokinesia of the left ventricular walls. A cardiac catheterization revealed coronary arteries without significant stenoses. Cardiac magnetic resonance imaging showed an ejection fraction of 27%, with global systolic dysfunction and absence of fibrosis, suggesting an idiopathic dilated cardiomyopathy. A 24-hour Holter was also performed, which did not show ventricular arrhythmias during the monitoring period.

After the patient was compensated with measures for heart failure at the institution, the patient underwent genetic testing for hereditary cardiomyopathies, and the genes listed in Table 1 were analyzed. The analysis was performed with genomic DNA extraction and fragmentation followed by identification, capture and enrichment of the regions of interest. The result of such examination revealed a likely pathogenic variant c.1595delIT in heterozygosity in the Filamin-C gene - *FLNC*. This gene has been associated with a number of heart diseases, such as: (1) familial hypertrophic cardiomyopathy, of undetermined inheritance; (2) familial restrictive cardiomyopathy,^1^ of autosomal dominant inheritance [OMIM: 617047]; (3) distal myopathy, of autosomal dominant inheritance [OMIM: 614065] and (4) myofibrillar myopathy, of autosomal dominant inheritance [OMIM: 609524].^2^ The identified variant is characterized by the deletion of a nucleotide that, predictably, leads to a change in the reading frame (frameshift) by promoting the replacement of the amino acid valine at codon 532 by a glycine, with consequent early stop of protein translation 16 positions ahead (p.(Val532Glyfs*16)) resulting in a truncated protein. The variant is absent in the population frequency databases (Exome Aggregation Consortium – ExAC and The Genome Aggregation Database - GnomAD),^3^ it has never been described in the medical literature and has never been observed in the ClinVar database.^4^ Filamin-C is a protein expressed mainly in cardiac and skeletal muscle, being encoded by the *FLNC* gene. The protein is responsible for the crosslinking of actin filaments in orthogonal networks in the cortical cytoplasm of cells and participates in the anchoring of membrane proteins to the actin cytoskeleton.^5^ Due to these functions and its expression predominantly in the cardiac muscle, the *FLNC* gene has been related to dilated or arrhythmogenic cardiomyopathies.^6^ According to the metrics available in the GnomAD database, the *FLNC* gene does not tolerate loss-of-function alterations. Furthermore, frameshift mutations have been related to arrhythmogenic/dilated cardiomyopathy diseases.^7^ According to the criteria of the American College of Medical Genetics and Genomics – ACMG,^8^ this found variant is classified as likely pathogenic.

**Table 1:** Analyzed Genetic Panel

ABCC9; ACTC1; ACTN2; ANK2; BAG3; BRAF; CALR3; CAV3; CBL; CRYAB; CSRP3; DES; DSC2; DSG2; DSP; DTNA; EMD; EYA4; FHL1; FKTN; FLNC; GAA; GLA; HRAS; JPH2; JUP; KRAS; LAMP2; LDB3; LMNA; MAP2K1; MAP2K2; MYBPC3; MYH6; MYH7; MYL2; MYL3; MYLK2; MYOT; MYOZ2; NEBL; NEXN; NRAS; PKP2; PLN; PRKAG2; PSEN1; PSEN2; PTPN11; RAF1; RBM20; RPSA; RYR2; SCN5A; SGCD; SHOC2; SLC25A4; SOS1; SPRED1; SYNE1; SYNE2; TAZ; TCAP; TGFB3; TMEM43; TMPO; TNNC1; TNNI3; TNNT2; TPM1; TRIM63; TTN; TTR; VCL.

The patient showed clinical improvement with the treatment and was discharged with furosemide, sacubitril-valsartan and carvedilol. Genetic counseling was recommended to the family, since first-degree relatives of individuals with heterozygous pathogenic variants of the *FLNC* gene have a 50% probability of being carriers of the same variant. Despite the risk of major ventricular arrhythmias, in this specific case, the patient chose not to receive an implantable cardioverter defibrillator (ICD) at first. Regarding this aspect of sudden cardiac death prevention, we recall that the DANISH^9^ study showed no benefits in terms of mortality reduction in patients with heart failure with reduced ejection fraction of nonischemic etiology. However, it is important to emphasize that, according to the guidelines of the Brazilian Society of Cardiology, patients with heart failure of non-ischemic etiology, with an ejection fraction ≤ 35% may have an indication for an ICD, including for primary prophylaxis (Class IIa).^10^ In addition, the Heart Rhythm Journal guidelines from 2019^11^ mention that in patients with arrhythmogenic cardiomyopathy linked to a mutation in the *FLNC* gene and ejection fraction < 45 %, the ICD implantation is a therapy to be considered (Class IIa/C). This would be a more specific recommendation, as it takes into account the genetic characteristic of the patient's pathology. We feel that the decision as to whether or not implant an ICD should take the literary evidence into account, but that it should also be individualized, always in compliance with the patient's wishes and their quality of life and life expectancy.

In view of this report, we consider the importance of establishing the etiology for cases of presumed idiopathic heart failure, including genetic research, as there is the possibility that a causal factor for a patient's disease may be found, a patient who often may be deprived of a more specific diagnosis. Evidently, there are certain difficulties in offering genetic screening, such as the low availability of the test in many places, high price and the lack of dissemination of genetic knowledge among general cardiologists. It should be noted that genetics is a field still undergoing great development, in which certainly many mutations and pathogenic variants still need to be catalogued, allowing much more precise advice to be provided to patients and their families, once the origin of the pathology in question has been determined.
